# Dermal Formulation Incorporating Isoconazole Nitrate Nanoparticles Offers High Absorption into Skin and Antimicrobial Effect Against *Candida albicans*

**DOI:** 10.3390/pharmaceutics17121519

**Published:** 2025-11-26

**Authors:** Ayako Harada, Rie Tanaka, Hiroko Otake, Masanori Yoshimura, Tae Wada, Tohru Nagahama, Noriaki Nagai

**Affiliations:** 1 ROHTO Pharmaceutical Co., Ltd., 1-8-1 Tatsuminishi, Ikuno-ku, Osaka 544-8666, Japan; ayakoh@rohto.co.jp (A.H.); yoshimura@rohto.co.jp (M.Y.); wadatae@rohto.co.jp (T.W.); nagahamatohru@rohto.co.jp (T.N.); 2 Faculty of Pharmacy, Kindai University, 3-4-1 Kowakae, Higashi-Osaka 577-8502, Japan; 2211710037t@kindai.ac.jp (R.T.); hotake@phar.kindai.ac.jp (H.O.)

**Keywords:** isoconazole nitrate, skin formulation, nanoparticle, carboxypolymethylene, drug delivery

## Abstract

**Background**: Isoconazole nitrate (ISN), an antifungal agent that inhibits ergosterol synthesis by blocking lanosterol 14α-demethylation, is widely used to treat candidiasis, and improving its skin retention and permeability can enhance its therapeutic efficacy. Therefore, we developed an ISN nanoparticle (ISN-NP) gel by wet-bead milling in the presence of methylcellulose (MC). **Methods**: These ISN nanoparticles were incorporated into a carboxypolymethylene hydrogel (Carbopol). The ISN concentration was measured using HPLC, and Wistar rats and *Candida albicans* were used to evaluate skin absorption and antifungal activity, respectively. **Results**: The ISN-NP gel exhibited a particle size distribution ranging from 60 to 220 nm, with the nanoparticles remaining stable. In addition, the ISN-NP gel demonstrated superior antifungal activity against *Candida albicans*. The Carbopol gel maintained appropriate viscosity and physical stability, and the ISN nanoparticles were released from the gel. Compared with microparticle-based gels (ISN-MP gels), the ISN-NP gel showed significantly enhanced drug release and transdermal permeation, with 1.54- and 1.7-fold increases, respectively. **Conclusions**: These findings indicate that incorporating ISN nanoparticles (nanocrystalline ISN) into a Carbopol-based gel matrix provides a promising strategy to enhance the topical delivery of this poorly water-soluble antifungal drug. Overall, this nanogel system represents a valuable platform for transdermal delivery in clinical applications.

## 1. Introduction

Isoconazole nitrate (ISN, 1-[2-(2,4-dichlorophenyl)-2-[(2,6-dichlorophenyl)methoxy]ethyl]-1H-imidazole mononitrate) is a compound that appears as a white to pale yellow powder. It demonstrates good solubility in methanol and dimethyl sulfoxide, limited solubility in ethanol, and is practically insoluble in water. As a member of the azole antifungal family, ISN exerts significant antifungal activity [1]. Azole antifungals are among the most commonly used agents in clinical mycology due to their broad spectrum of activity. These agents primarily function by targeting and inhibiting fungal cytochrome P450-dependent 14α-demethylase. ISN exhibits extensive antimicrobial efficacy, not only against dermatophytes and Candida and Aspergillus species but also against certain Gram-positive bacteria, including strains of Staphylococcus and Streptococcus [2]. Clinically, ISN is indicated for the management of infections such as dermatophytosis and both cutaneous and vaginal candidiasis [3].

The predominant antifungal mechanism of ISN involves interrupting ergosterol production by inhibiting lanosterol 14α-demethylation. This disruption prevents the normal biosynthesis of ergosterol, leading to the accumulation of 14α-methylsterols—intermediates that compromise membrane function, enhance permeability, and ultimately contribute to fungal cell demise [3,4]. With prolonged exposure, ISN may also exhibit fungicidal activity via a distinct mechanism that involves rapid, direct damage to the fungal cell membrane, independent of ergosterol pathway interference [5]. The antifungal effects of ISN have been attributed to its interaction with oxidized flavohemoglobin, potentially leading to the degradation of enzyme-generated superoxide, a marker of oxidative stress [6]. Due to these combined actions, ISN is regarded as an effective therapeutic agent with both fungistatic and fungicidal properties, making it particularly suited for treating superficial fungal infections [2].

Drug delivery through the skin can occur via three principal pathways [7,8,9]. The first is the transcellular (or intracellular) route, which enables hydrophilic compounds to diffuse directly through corneocytes [7,8,9]. Although this pathway is less efficient in the stratum corneum (SC) due to its low water content (approximately 13%), it becomes more relevant in the underlying dermal layers once the SC barrier is overcome. The second route is the intercellular (or paracellular) pathway, where substances traverse the lipid-rich spaces between adjacent cells. This mechanism is considered the primary route for lipophilic or hydrophobic compounds. The third is follicular transport, which involves the penetration of molecules through hair follicles. This pathway is particularly important for delivering polar, hydrophilic agents, high-molecular-weight compounds, and nanoparticulate drug carriers.

Despite the formidable barrier properties of the skin, submicron nanoparticles have demonstrated the ability to penetrate its deeper layers. These nanosized carriers also exhibit an enhanced affinity for cellular membranes, which may prolong their retention at the application site [10]. Based on these characteristics, a wide range of nanocarrier systems—such as nanocrystals, solid lipid nanoparticles, nanoemulsions, niosomes, ethosomes, transfersomes, polymeric nanoparticles, and liposomes—have been developed to optimize transdermal and topical drug delivery [11,12,13]. Nanoparticles containing nanocrystals, defined as crystalline drug particles with diameters typically between 100 and 1000 nm, have recently gained attention as effective carriers for dermal administration. Their small size increases the drug’s saturation solubility and dissolution rate, largely due to the increased surface area. This enhanced solubility generates a steeper concentration gradient across the skin barrier, promoting greater passive diffusion [14,15]. Moreover, nanoparticles of this size have been shown to accumulate preferentially in hair follicles, facilitating follicular targeting. They also exhibit strong adhesive interactions with skin cells, further enhancing the formulation’s residence time on the skin surface [10]. Compared with other nanocarriers, nanoparticles, such as nanocrystals, offer additional benefits, including high drug loading, minimal surfactant requirements, cost-effectiveness, reproducibility, and scalability [16]. Their prolonged skin retention time and enhanced penetration are particularly advantageous for treating fungal infections [17]. In fact, previous studies have reported that nanogels loaded with clotrimazole nanocrystals achieved the intended goal of retaining clotrimazole within the skin layers, thereby exerting prolonged therapeutic effects. This approach was further shown to improve patient compliance in individuals with candidiasis and contribute to the short-term treatment of cutaneous candidiasis [18]. Regarding changes in absorption characteristics via control of drug crystals, it has been reported that a conazole salt cocrystal (econazolium–gallic acid–econazole) exhibits dual optimization of physicochemical properties and antifungal activity. These findings highlight the potential of formulation designs that preserve the crystalline state [19]. Considering these benefits, this study formulated a nanosuspension based on ISN crystalline and incorporated it into gel matrices for dermal delivery.

In this study, we explored the production of ISN nanoparticles with particle sizes below 200 nm using bead milling. In addition, we evaluated the effectiveness of incorporating these nanoparticles into Carbopol-based hydrogels (ISN-NP gels) for dermal application, with a focus on enhancing the transdermal absorption of ISN.

## 2. Materials and Methods

### 2.1. Animals

Seven-week-old male Wistar rats were obtained from Kiwa Laboratory Animals Co., Ltd. (Wakayama, Japan). The animals were maintained at a constant temperature of 25 °C with unrestricted access to drinking water and a standard CE-2 diet (Clea Japan Inc., Tokyo, Japan). All procedures involving animals were conducted in accordance with the ethical guidelines established by Kindai University, the Japanese Pharmacological Society, and the U.S. National Institutes of Health (NIH). The study protocol received institutional approval from Kindai University on 1 April 2022 (approval no. KAPS-2022-017). The experimental procedures were performed in compliance with the ARRIVE guidelines and the 2020 AVMA Guidelines for the Euthanasia of Animals. Euthanasia was conducted via intraperitoneal injection of pentobarbital at a dose of 200 mg/kg.

### 2.2. Chemicals

We used commercially available chemicals of the highest purity. Briefly, ISN was purchased from ERREGIERRE S.p.A. (San Paolo D’Argon, Italy), and Carbopol (carboxypolymethylene, Carbopol^®^ 934) was obtained from Serva (Heidelberg, Germany). Methylcellulose (MC) was provided by Shin-Etsu Chemical Co., Ltd. (Tokyo, Japan), and membranes with a pore size of 450 nm were purchased from GVS Japan (Tokyo, Japan) for the drug release study. Pentobarbital was obtained from Tokyo Chemical Industry Co., Ltd. (Tokyo, Japan).

### 2.3. Design of Carbopol Gel Incorporating ISN Nanoparticles

Carbopol-based gels containing either micro- or nanosized ISN were formulated following established protocols from our previous studies [20,21,22]. In brief, nanoparticle formulations were prepared by bead milling using a rotation/revolution-type mixing system (Nano Pulverizer NP-100, THINKY CORPORATION, Tokyo, Japan). A suspension composed of 1% ISN and 4% MC in purified water was combined with 0.1 mm zirconia beads in a sealed tube and processed at 2000 rpm for 3 min per cycle, repeated 10 times, under cooling at 4 °C. The resulting milled dispersion was designated as milled-ISN. This milled ISN dispersion was converted into a gel by incorporating Carbopol pre-dissolved in distilled water, yielding the ISN-NP gel used in this study. For comparison, an ISN microparticle-containing gel (ISN-MP gel) was prepared by dispersing unprocessed ISN crystals and MC in distilled water, followed by mixing with a Carbopol^®^ 934 solution to form a gel. Both the ISN-NP and ISN-MP gels contained 1% ISN, 4% MC, and 2% Carbopol (*w*/*w* in distilled water). Both formulations met the criteria of the Japanese Pharmacopoeia Uniformity of Dosage Units test, with deviation values within 1%. The zeta potential was obtained through the use of a Micro-Electrophoresis Zeta Potential Analyzer model 502 (Nihon Rufuto Co., Ltd., Tokyo, Japan).

### 2.4. Particle Characteristics of ISN Gels

Particle size analysis of the ISN gels was conducted by diluting 0.3 g of the gel in 100 mL of distilled water, followed by stirring for 10 min. The resulting dispersion was subjected to particle size measurement using two analytical systems: the SALD-7100 (Shimadzu Corporation, Kyoto, Japan) and the NanoSight LM10 (Quantum Design Japan, Inc., Tokyo, Japan). For the SALD-7100 measurements, the scattering intensity was maintained within the optimal range of 40–60%, and the refractive index was set at 1.60 ± 0.10 i. For nanoparticle tracking analysis using the NanoSight LM10, the conditions included a blue laser with a wavelength of 405 nm, a recording time of 60 s, and a medium viscosity of 1.27 mPa·s.

### 2.5. SEM and SPM Images of ISN-NP Gel

For morphological analysis, 0.3 g of the ISN gel was diluted with 100 mL of distilled water and stirred for 10 min. The resulting dispersion was then used for imaging with scanning electron microscopy (SEM) and scanning probe microscopy (SPM). SEM observations were performed using a NeoScope™ JCM-7000 (JEOL Ltd., Tokyo, Japan), and SPM imaging was conducted with the SPM-9700 system (Shimadzu Corporation, Kyoto, Japan). The results were presented as composite images combining the phase and height topographies of the ISN particles.

### 2.6. Measurement of ISN Concentration

To quantify ISN concentrations, 50 µL of each sample was mixed with 100 µL of methanol and subjected to HPLC analysis using a Shimadzu LC-20AT liquid chromatography system equipped with a CTO-20A column oven (Shimadzu Corp., Kyoto, Japan). An aliquot of 10 µL was introduced into the system via an SIL-20AC autosampler (Shimadzu Corp., Kyoto, Japan). Chromatographic separation was achieved using an L-column ODS (4.6 mm internal diameter × 150 mm length, 5 µm particle size; GL Sciences Inc., Tokyo, Japan) maintained at 40 °C. The mobile phase consisted of methanol and a 0.2% ammonium acetate aqueous solution in a 41:59 (*v*/*v*) ratio, delivered at a flow rate of 1.0 mL/min. Detection was performed at a wavelength of 230 nm. The linearity, R^2^ value, LOD, LOQ, and LOD/LOQ were y = 2.6635x + 0.0359, 0.9991, 0.19 µg/mL, 0.5 µg/mL, and 0.38, respectively.

### 2.7. Viscosity and Spreadability Tests of ISN Gels

The viscosity of the ISN gels was evaluated using a Brookfield digital viscometer equipped with a CPZ-52Z cone-plate spindle (Brookfield Engineering Laboratories, Inc., Middleboro, MA, USA). Measurements were performed at 60 rpm for 3.5 min at a controlled temperature of 20 °C. The fluidity of ISN gels was evaluated using spread meter No. 419 (RIGO Co., Ltd., Tokyo, Japan), and the spreadability and yield stress were determined. In this test, 1 g of the ointment was applied, and the measurements were performed at 20 °C.

### 2.8. Drug Solubility of ISN Gels

A 0.3 g portion of each ISN gel was mixed with 10 mL of distilled water and stirred continuously for 10 min. The resulting dispersion was then subjected to ultracentrifugation at 100,000× *g* using an Optima™ MAX-XP ultracentrifuge (Beckman Coulter, Osaka, Japan) to separate the solubilized ISN from the undissolved fraction. The concentration of the solubilized ISN, representing its solubility, was subsequently quantified by HPLC under the conditions described previously.

### 2.9. Antimicrobial Effect of ISN in Candida albicans

Antifungal activity was evaluated using the agar well diffusion method. Sabouraud Dextrose Agar (SDA; Nissui Pharmaceutical Co., Ltd., Tokyo, Japan) was used, consisting of a base layer (10 mL) and a seed overlay (5 mL) inoculated with *Candida albicans* (ATCC 10231/NBRC 1594) at a final density of 1 × 10^6^ CFU/mL. Wells (6 mm diameter) were aseptically prepared, and 100 µL of the test sample was applied. The plates were incubated at 25 °C for 48 h, and inhibition zones were measured in millimeters using a Protocol 3 colony counter (Synbiosis, London, UK). In this method, when an antifungal agent is applied, a circular inhibition zone (area where no fungal growth occurs) is formed around the center of the treated region. In this study, the maximum diameter and the area of the inhibition zone were measured, and these parameters were used to assess antifungal activity.

### 2.10. Drug Release from ISN Gel

The in vitro release profile of ISN from the gels was evaluated based on a previously reported method [23], with slight modifications. A Franz diffusion cell equipped with a 12.2 mL receptor compartment was employed and filled with 0.2 mM phosphate buffer (pH 7.2) as the receptor medium. A membrane with a pore size of 0.45 μm was mounted between the donor and receptor compartments. Subsequently, 0.3 g of the ISN gel was evenly applied to the membrane surface (diffusion area: 2 cm^2^) and incubated at 37 °C with a stirring speed of 300 rpm (diameter 1 cm, crosshead stirring bar) for 24 h. At designated time intervals (0–24 h), 100 μL samples were withdrawn from the receptor compartment. The concentration of ISN and its particle size distribution were analyzed using HPLC and a NanoSight LM10, respectively. The area under the concentration–time curve for the released ISN (*AUC*_release_) was computed using the trapezoidal rule through to final sampling at 24 h. Moreover, the drug release rate constant (*k*_R_) was calculated from the values obtained up to 6 h after the start of the experiment, during which a linear release profile was observed, using the zero-order equation.

### 2.11. In Vitro Transdermal Penetration of ISN Gel

Full-thickness abdominal skin, including the SC, epidermis, and dermis, was harvested from fourteen seven-week-old Wistar rats, which were randomly divided into two groups (*n* = 7 per group). Hair was removed by shaving 24 h prior to the experiment. The animals were euthanized via intraperitoneal administration of pentobarbital (200 mg/kg), and the isolated skin samples were fitted onto Franz diffusion cells. The receptor compartment (12.2 mL) was filled with 0.2 mM phosphate buffer (pH 7.2) and maintained at a constant temperature of 37 °C. A total of 0.3 g of ISN gel was applied evenly to the skin surface (diffusion area: 2 cm^2^) and incubated at 37 °C with a stirring speed of 300 rpm (diameter 1 cm, crosshead stirring bar). At specific time points (1, 2, 6, and 24 h), 100 µL samples were withdrawn from the receptor chamber for analysis using HPLC and NanoSight LM10. The cumulative transdermal drug absorption, represented by the area under the concentration–time curve (*AUC*_penetration_), was estimated using the trapezoidal integration method. Moreover, the drug penetration rate constant (*k*_P_) was calculated from the values obtained up to 24 h after the start of the experiment according to the first-order equation.

### 2.12. Statistical Analysis

All data are presented as the mean ± standard error of the mean (S.E.). Statistical evaluations were conducted using JMP software version 5.1 (SAS Institute, Cary, NC, USA). Comparisons between two groups were conducted using Student’s *t*-test, while one-way analysis of variance (ANOVA) was used for multiple group comparisons, followed by the Tukey–Kramer post hoc test. A *p*-value of less than 0.05 was considered to indicate statistical significance.

## 3. Results

### 3.1. Characteristics of Dermal Formulation Incorporating ISN Nanoparticles

**Figure 1:**
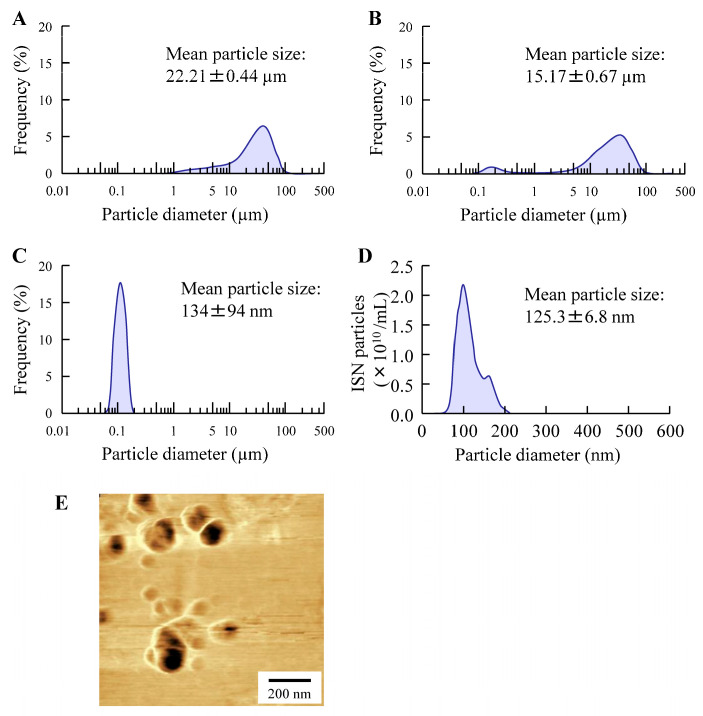
Particle size distribution of ISN with and without bead milling treatment. (**A**) Particle size of ISN powder, determined using SALD-7100 (**A**). (**B**,**C**) Particle size of ISN exposed (milled-ISN) or not (ISN-sus) to bead milling with additives, determined using SALD-7100. (**D**) Particle size of milled-REB with additives, determined using NanoSight LM10. (**E**) SPM image of milled-REB with additives. The bead milling treatment decreased the mean particle size, and the particle size of the milled-ISN was in the range of 50–220 nm.

**Figure 2:**
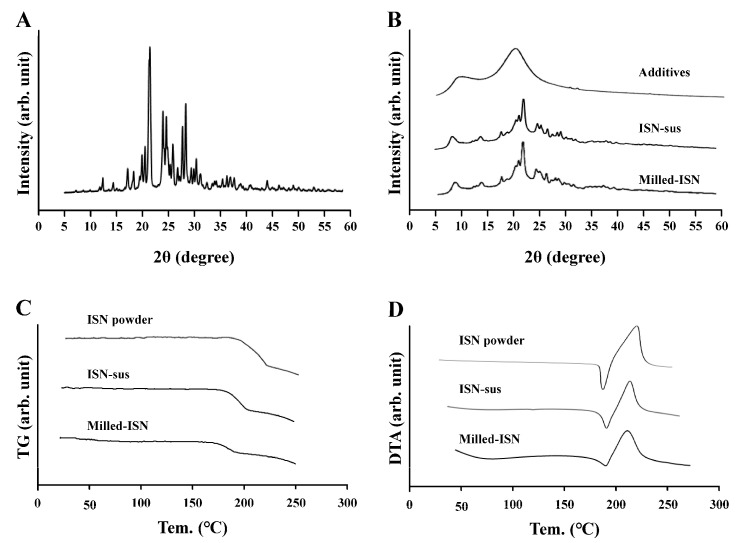
XRD and TG-DTA patterns of ISN exposed (milled-ISN) or not (ISN-sus) to bead milling. (**A**) XRD pattern of ISN powder. (**B**) Changes in XRD patterns of vehicle, ISN-sus, and milled-ISN with additives. (**C**) Changes in TG patterns of ISN powder, ISN-sus, and milled-ISN with additives. (**D**) Changes in DTA patterns of ISN powder, ISN-sus, and milled-ISN with additives. The same peak was detected between ISN-sus and milled-ISN, and no difference in TG-DTA pattern was observed in ISN with and without the bead milling treatment.

**Figure 3:**
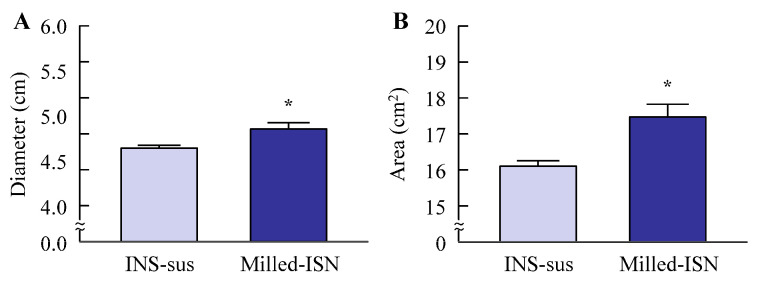
Antimicrobial effect of ISN-NP gels on *Candida albicans*. (**A**,**B**) The maximum diameter (**A**) and area (**B**) of the inhibition zone (non-growth region) in the drug-treated area determined using the agar well diffusion method. In this method, when an antifungal agent is applied, a circular inhibition zone (an area where no fungal growth occurs) forms around the center of the treated region. In this study, the maximum diameter (**A**) and the area (**B**) of the inhibition zone were measured, and these parameters were used to assess antifungal activity. *n* = 5. * *p* < 0.05 vs. ISN-MP gel for each category. The antimicrobial effect of the ISN-NP gel on *Candida albicans* was higher than that of the ISN-MP gel.

**Figure 4:**
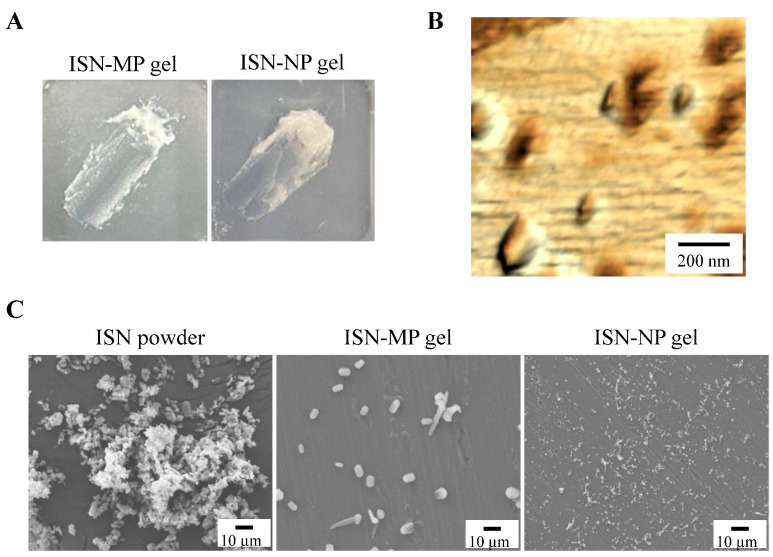
Images of ISN-NP gel. (**A**) Photographs of ISN-MP and ISN-NP gels (size: 2 cm × 2 cm). (**B**) SPM images of ISN-NP gel. (**C**) SEM images of ISN powder, ISN-MP, and ISN-NP gels. The particles of ISN in the ISN-NP gel remained nano-sized.

**Figure 5:**
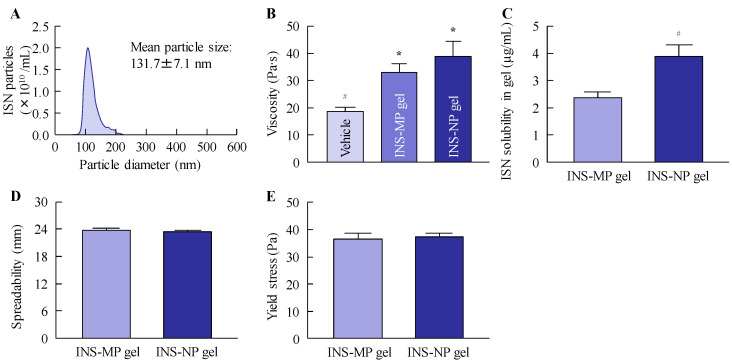
Characteristics of ISN-NP gel. (**A**) Particle distribution of ISN in ISN-NP gel. Changes in viscosity (**B**), solubility (**C**), spreadability (**D**), and yield stress (**E**) of ISN-MP and ISN-NP gels. *n* = 5. * *p* < 0.05 vs. vehicle for each category. ^#^ *p* < 0.05 vs. ISN-MP gel for each category. The particle size of ISN in the ISN-NP gel is in the range of 60–220 nm. Although spreadability and yield stress were similar for both ISN gels, the viscosity, and solubility of ISN-NP gel were higher than those of ISN-MP gel.

Figure 1 shows changes in the ISN particle size following the bead milling treatment. The mean ISN particle size was 15.17 ± 0.67 µm (Figure 1B), and the bead milling treatment decreased the particle size. The mean ISN particle size was 50–220 nm (Figure 1C,D). ISN particles smaller than 200 nm were also identified in the SPM images, and their shapes were nearly spherical (Figure 1E). The zeta potentials of INS-sus and milled-ISN were 6.8 ± 0.8 mV and 7.3 ± 0.9 mV, respectively. Figure 2 shows the XRD and TG-DTA patterns. The ISN-sus and milled-ISN had similar peak XRD patterns (Figure 2B). In the TG-DTA data, thermal analysis revealed a significant weight loss above approximately 180 °C. A corresponding endothermic peak was also observed at a similar temperature in the DTA profile. Given that the reported melting point of ISN is 178 °C, these changes in TG-DTA patterns are suggested to be associated with the melting of the compound. These thermal behaviors were consistent across bead milling treatments. Figure 3 shows the therapeutic effect of ISN gel for *Candida albicans*. Both the INS-sus and milled-ISN demonstrated antifungal activity against *Candida albicans*. Notably, the antifungal activity of the milled-ISN was significantly higher than that of ISN-sus. Subsequently, gels containing ISN, with or without bead milling, were prepared using Carbopol as the polymeric matrix. Figure 4 shows the digital, SPM, and SEM images of these ISN-MP and ISN-NP gels, and Figure 5 shows the particle distribution, viscosity, and solubility of the drug in the ISN-MP and ISN-NP gels. The ISN maintained its nanoscale size in the gel, with an average particle diameter of 131.7 ± 7.1 nm. The incorporation of ISN increased viscosity; specifically, the viscosities of the ISN-MP and ISN-NP gels were 1.8- and 2.1-fold higher than that of the vehicle, respectively. Furthermore, the solubility of ISN in the gel was 1.6 times higher in the ISN-NP gel compared with the ISN-MP gel. On the other hand, the spreadability and yield stress were not different between the ISN-MP and ISN-NP gels.

### 3.2. Drug Absorption Behavior of ISN Gel in Rat Skin

**Figure 6:**
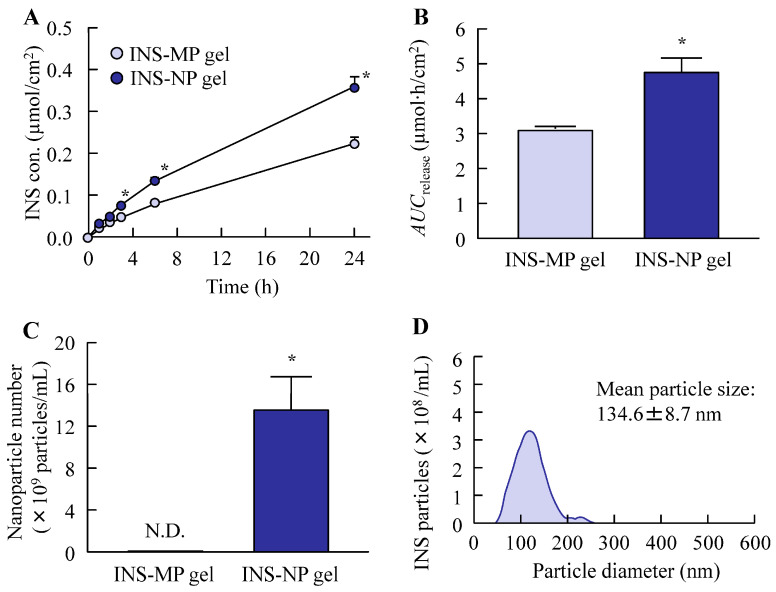
Release behavior of INS from ISN-MP and ISN-NP gels through a 450 nm pore membrane. Release behavior (**A**) and *AUC*_release_ (**B**) of ISN from ISN-MP and ISN-NP gels. Number (**C**) and size distribution (**D**) of ISN nanoparticles in the reservoir chamber 24 h after the application of ISN-NP gel. *n* = 7. N.D., not detectable. * *p* < 0.05 vs. ISN-MP gel for each category. The *AUC*_release_ in the ISN-NP gel was 1.54-fold that in the ISN-MP gel, and ISN nanoparticles were detected in the reservoir chamber after the application of the ISN-NP gel.

**Figure 7:**
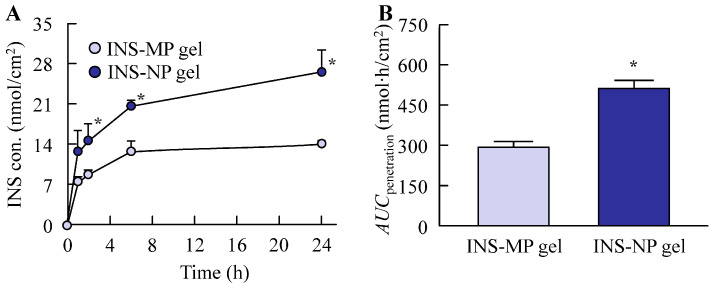
Changes in permeability (**A**) and *AUC*_penetration_ (**B**) of ISN-MP and ISN-NP gels applied to rat skin. *n* = 7. * *p* < 0.05 vs. ISN-MP gel for each category. The *AUC*_penetration_ of the ISN-NP gel was 1.68-fold that of the ISN-MP gel. In contrast to the release behavior results, ISN nanoparticles were not detected in the reservoir chamber 24 h after the application of the ISN-NP gel.

Figure 6 shows the drug release profiles of the ISN-MP and ISN-NP gels, as well as the physical state of the ISN after its release. Drug release was significantly higher from the ISN-NP gel than from the ISN-MP gel, with the *AUC*_release_ being 1.5 times greater. Moreover, the *k*_R_ values for the ISN-MP and ISN-NP gels were 0.014 ± 0.002 µmol/cm^2^/h and 0.022 ± 0.003 µmol/cm^2^/h, respectively, with the ISN-NP gel showing a higher value. Although the ISN released from the ISN-MP gel was completely dissolved, the ISN released from the ISN-NP gel remained in a nanosized state, with an average particle diameter of 134.6 ± 8.7 nm. In addition, XRD and TG-DTA analyses of the ISN nanoparticles released from the gel revealed that they possessed the same crystal form and melting point as the nanoparticles prepared initially, which exhibited strong antifungal activity (Figure S1). Figure 7 shows the penetration of ISN into rat skin to which the ISN-MP and ISN-NP gels were applied. Similar to the results observed in the in vitro release study (Figure 6), the skin penetration of ISN was enhanced by the bead milling treatment. The *AUC*_penetration_ of the ISN-NP gel was approximately 1.7 times higher than that of the ISN-MP gel. Moreover, the ISN-NP gel penetrated the skin more rapidly; the amount of drug permeated at 1 h post-application was about 1.9 times greater than that of the ISN-MP gel, and the *k*_P_ values of the ISN-NP gel (0.96 ± 0.09 h^−1^) were significantly higher than those of the ISN-MP gel (0.58 ± 0.08 h^−1^).

## 4. Discussion

ISN acts by blocking lanosterol 14α-demethylation, a key step in the biosynthetic pathway of ergosterol in fungal cell membranes, and is commonly used to manage infections, such as cutaneous and vaginal candidiasis [3]. In this context, enhancing its cutaneous retention and permeability across the skin barrier may offer improved therapeutic benefits. Therefore, in this study, we developed a Carbopol-based gel incorporating ISN nanoparticles (ISN-NP gel) to evaluate its potential to facilitate transdermal delivery. In conclusion, we found that bead milling successfully produced nanoparticles with sizes ranging from 50 to 220 nm, maintaining their nanoscale integrity within the gel matrix. Furthermore, our findings indicate that the skin penetration of the ISN nanoparticles released from the gel was significantly improved, highlighting the potential of this delivery system for targeted antifungal therapy.

Initially, we focused on formulating the ISN-NP gel by applying nanoparticle-engineering strategies. Nanoparticle (nanocrystal) fabrication typically relies on bottom-up, top-down, or hybrid techniques. Among these options, the top-down approach—characterized by its use of intensive mechanical energy—is widely adopted and includes techniques such as wet media milling and high-pressure homogenization. These processes reduce bulk drug crystals of micrometer dimensions into nanosized particles through mechanical shear and collision forces in the presence of a stabilizing aqueous medium [24]. We previously reported that drug nanoparticles with a diameter of less than 200 nm can be obtained via bead milling with appropriate additives [20,22]. Moreover, we showed that MC enhanced crushing efficiency and was a suitable additive for the production of the drug nanoparticles [21]. In this study, we successfully prepared ISN nanoparticles (nanocrystals) that maintained their crystalline form by employing a wet bead milling method, using MC and HPβCD as stabilizing additives (Figure 1 and Figure 2). Subsequently, we evaluated potential alterations in ISN’s antifungal activity against *Candida albicans* before and after bead milling. The results demonstrated that ISN retained its strong antifungal efficacy irrespective of the nanocrystallization treatment, indicating that mechanical processing did not compromise its therapeutic potency. Interestingly, the ISN processed by bead milling to achieve nanoscale dimensions exhibited greater antifungal efficacy than its unprocessed counterpart (Figure 3). It is well documented that nanoparticles exhibit increased adhesion to cellular membranes [23,25]. This increased interaction with the microbial surface may partly explain the improved antifungal effects observed with the nanonized ISN. However, further studies are warranted to clarify these mechanisms.

To formulate these ISN nanoparticles into a stable, skin-applicable form, we focused on incorporating them into a hydrogel base. Carbopol, a cross-linked polyacrylic acid polymer, is widely used as a hydrogel matrix in topical formulations due to its excellent swelling capacity, biocompatibility, and ability to form transparent, high-viscosity gels at low concentrations. Its thixotropic properties and pH-sensitive gelation behavior make it particularly suitable for dermal drug delivery systems [25]. By utilizing Carbopol as the hydrogel base, we successfully prepared an ISN-NP gel in which ISN was uniformly dispersed and retained in its nanoparticulate form, without aggregation or sedimentation. The skin permeation properties of the ISN-NP gel were subsequently investigated. It is well known that the skin permeation of a gel is influenced by both the release of the drug from the base matrix and the drug’s ability to penetrate the skin. Therefore, we examined drug release from the ISN gel before studying its skin permeation. As shown in Figure 6A,B, the ISN-NP gel released significantly more of the drug compared with the ISN-MP gel. Furthermore, while only dissolved ISN was released from the ISN-MP gel, both dissolved ISN and solid ISN nanoparticles were released from the ISN-NP gel (Figure 6C,D). Moreover, the ISN nanoparticles released from the gel showed the same crystal form and melting point as the nanoparticles prepared initially, and they exhibited strong antifungal activity (Figure 3 and Figure S1). These findings suggest that reducing particle size improves solubility and increases surface area, and that the Carbopol matrix enhances viscoelastic stability and prolongs drug release. In addition, the ISN-NP gel may be a promising formulation for the effective delivery of solid nanoparticles into the skin. Next, we assessed the skin permeation of the ISN-NP gel.

Similar to the enhanced release of ISN from the base matrix, the transdermal permeability of ISN was also significantly higher with the ISN-NP gel compared with the ISN-MP gel (Figure 7). Notably, while no detectable skin permeation was observed with the ISN-MP gel between 8 h and 24 h after application, the ISN-NP gel exhibited sustained drug permeation beyond 8 h post-application (Figure 7A). These results indicate that the ISN-NP gel demonstrates both rapid initial absorption and prolonged transdermal drug delivery. It is well known that ISN is effective against *Candida albicans*. As shown earlier, the ISN nanoparticles we used exhibited high efficacy against *Candida albicans* (Figure 3), and topical application of the ISN-NP gel resulted in ISN penetration into the deeper layers of the skin (Figure 7). Furthermore, the ISN nanoparticles released from the gel were confirmed to retain the same crystalline structure as that of ISN used in the antifungal test (Figure S1). Therefore, we consider that the increased drug concentration achieved with the ISN-NP gel contributes to enhanced antifungal activity. On the other hand, to achieve “broad-spectrum activity”, a more extensive microbiological evaluation against dermatophytes and filamentous fungi (e.g., Trichophyton rubrum, Aspergillus niger) is important. To further clarify the potential utility of this formulation, future studies should address the aforementioned issues.

It is important to elucidate the mechanisms underlying the enhanced skin permeation of ISN when applied as the ISN-NP gel. The enhanced skin permeation observed with the ISN-NP gel may be attributed to several factors inherent to the nanoparticle formulation. First, the reduced size of the ISN nanoparticles likely increases the surface area available for dissolution, facilitating a higher concentration gradient across the SC [14,15]. Second, it is possible that the nanoparticles can penetrate the skin via appendageal routes, such as hair follicles and sweat glands, which are known to serve as alternative pathways for transdermal drug delivery [10]. Third, the prolonged presence of nanoparticles at the skin surface may serve as a reservoir, enabling sustained release and absorption over time [17]. Additionally, a reduction in particle size allows the nanoparticles to penetrate the stratum corneum. In contrast, because nanoparticles are larger than solvated molecules in solution, their diffusion in the skin tissue is reduced, which may contribute to increased and sustained drug concentrations within local tissues. These combined effects may account for both the rapid initial absorption and extended transdermal delivery profile observed with the ISN-NP gel. Although we proposed the transfollicular and intercellular transport pathways, these remain speculative at present. Therefore, further investigations are required to elucidate the hypothesized mechanisms underlying these enhanced skin permeation properties. To clarify the mechanisms underlying nanoparticle-mediated transdermal delivery, we are currently conducting additional investigations using follicular uptake analysis and confocal laser scanning microscopy. In addition, it is important to conduct imaging studies (e.g., confocal microscopy or fluorescence labeling) or histological localization studies to verify nanoparticle penetration. Furthermore, future investigations are planned to evaluate the antimicrobial activity and duration of action of the ISN-NP gel, using *Candida albicans* as a model organism.

## 5. Conclusions

**Figure 8:**
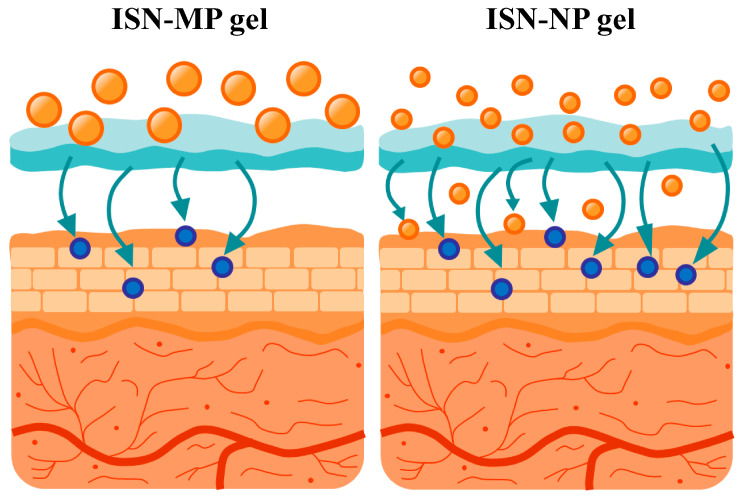
Scheme for absorption after application with ISN-NP gel. The ISN nanoparticles released from the ISN-NP gel enhance the dermal absorption of ISN, thereby increasing its therapeutic (antimicrobial) effect.

We successfully designed an ISN nanoparticle-incorporating hydrogel (ISN-NP gel) via bead milling, using MC as a stabilizer and Carbopol as a gel base. The ISN-NP gel exhibited significantly improved drug release and skin permeation compared with ISN-MP gels, maintaining ISN in its crystalline nanoparticulate form (Figure 8). These enhancements suggest nanoparticle-mediated delivery via possible transfollicular pathways. Our ongoing mechanistic studies will further clarify this result. This approach offers a promising platform for enhancing the topical delivery of poorly soluble drugs, such as ISN, and may support future advances in transdermal nanomedicine targeting *Candida albicans*. On the other hand, the aim of this study was to investigate, at a basic research level, whether nanosizing the antifungal drug ISN enhanced its skin permeability. For this purpose, Carbopol was selected as a representative gel base, and an ISN-MP gel containing microsized particles was used as a control to isolate the effect of particle size. The results demonstrated, for the first time, that this technique enables the delivery of an antifungal agent into the skin. However, to enhance commercialization potential, further studies are required to identify more suitable gel bases or excipients that can improve the release and long-term stability of ISN-NPs, as no aggregation of nanoparticles or drug degradation was observed after one month of storage under different conditions (4, 25, and 40 °C), but the viscosity increased over time due to water evaporation in this formulation (Figure S2). In addition, for practical application and industrial development, it is essential to obtain data comparing the formulation characteristics and therapeutic efficacy with those of a commercial ISN cream or marketed antifungal formulation, as well as data demonstrating consistent batch-to-batch uniformity in particle size, rheological profiling, and drug content. Additional investigations are therefore required to address these aspects.

## Data Availability

The raw data supporting the conclusions of this article will be made available by the authors upon request.

## Supplementary Materials

The following supporting information can be downloaded at: https://www.mdpi.com/article/10.3390/pharmaceutics17121519/s1, Figure S1: XRD (A) and TG-DTA patterns (B) of ISN released from the ISN-NP gel in the release test through a 450 nm pore membrane of Figure 6. Figure S2: Stability testing of ISN-NP gel under different conditions (4 °C, 25 °C, and 40 °C) for 1 month. (A–C) SEM images, ISN content, and Viscosity of ISN-NP gel after 1 month of storage under 4 °C, 25 °C, and 40 °C condition.

## Abbreviations

*AUC*_penetration_	Area under the concentration–time curve for skin-penetrated isoconazole nitrate
*AUC*_release_	Area under the concentration–time curve for released isoconazole nitrate
Carbopol	Carboxypolymethylene
ISN	Isoconazole nitrate
ISN-MP gel	Isoconazole nitrate microparticle-containing gel
ISN-NP gel	Isoconazole nitrate nanoparticle-containing gel
*k*_P_	Drug penetration rate constant
*k*_R_	Drug release rate constant
MC	Methylcellulose
SC	Stratum corneum
S.E.	Standard error
SEM	Scanning Electron Microscopy
SPM	Scanning Probe Microscopy
