# Supplementary figures and images for: Dermal Formulation Incorporating Isoconazole Nitrate Nanoparticles Offers High Absorption into Skin and Antimicrobial Effect Against Candida albicans
Source: Pharmaceutics. 2025 Nov 26;17(12):1519. doi: 10.3390/pharmaceutics17121519 (PMC12736124; doi:10.3390/pharmaceutics17121519)

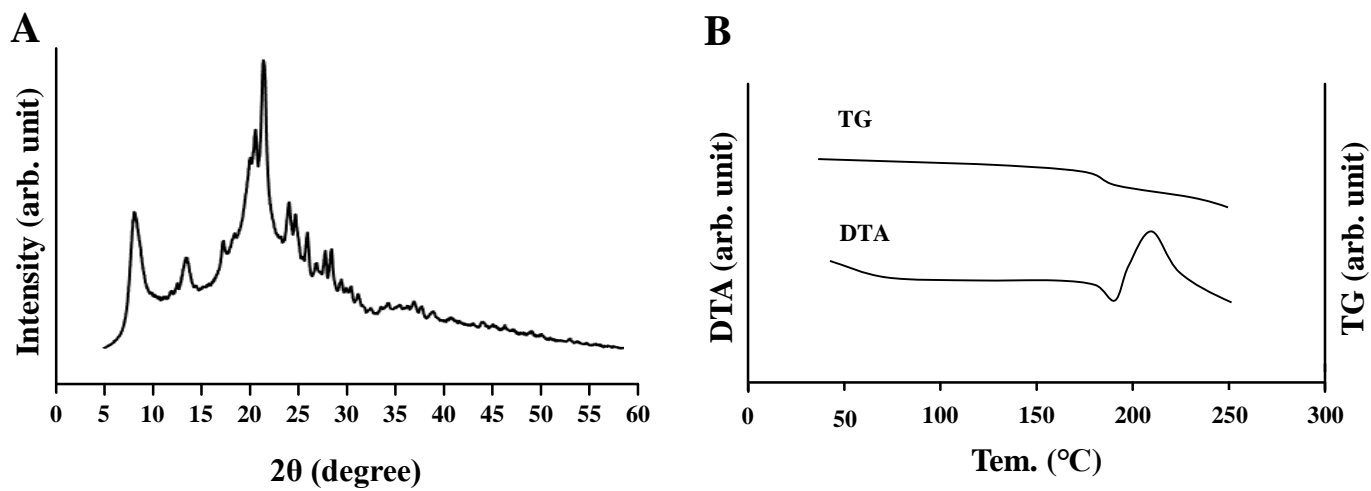

**Figure S1**

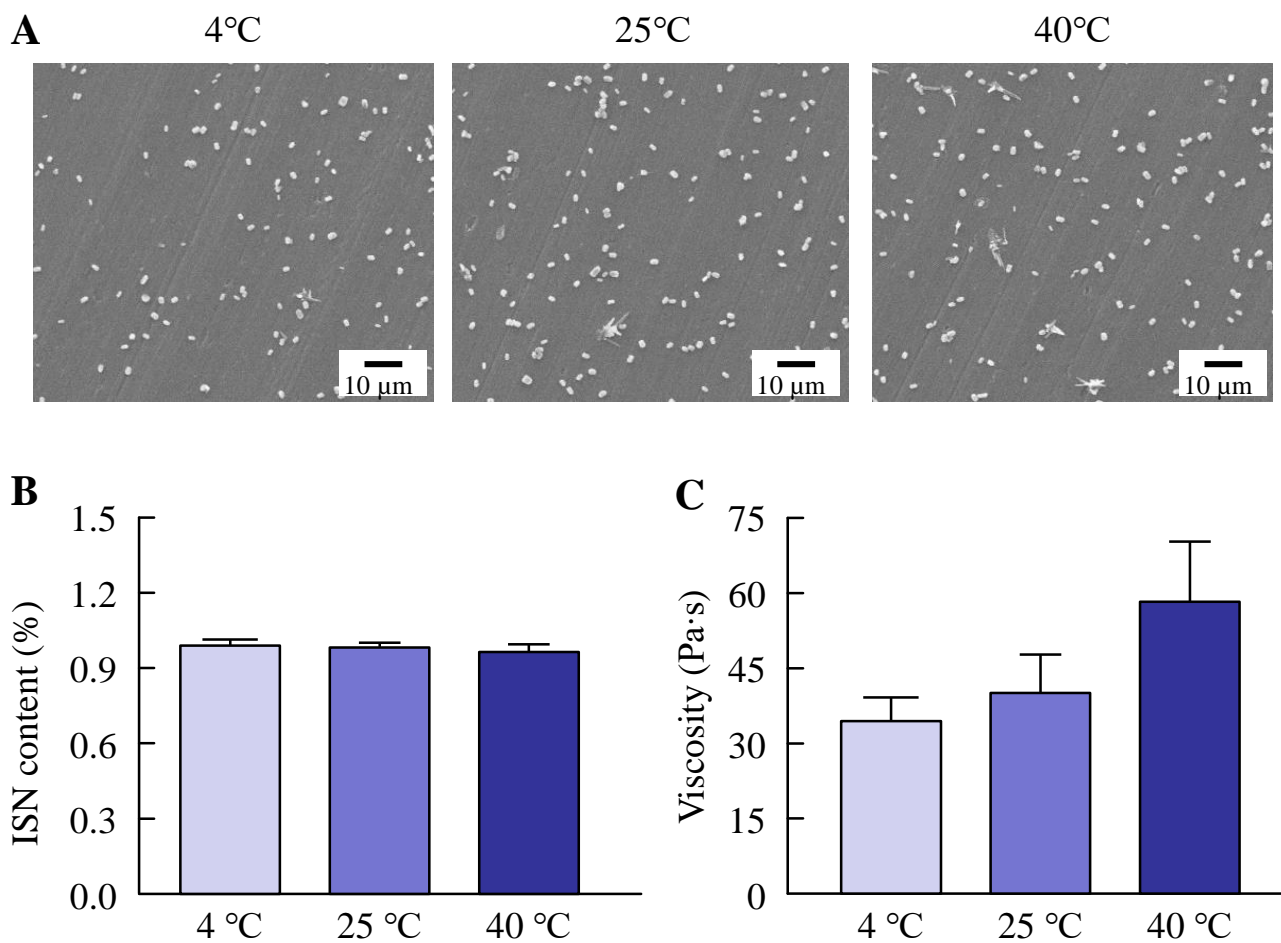

**Figure S2**

Supplement: Supplementary file 1 [file pharmaceutics-17-01519-s001.zip › pharmaceutics-3977349-supplementary.pdf]
